# A Dynamical Systems Hypothesis of Schizophrenia

**DOI:** 10.1371/journal.pcbi.0030228

**Published:** 2007-11-09

**Authors:** Marco Loh, Edmund T Rolls, Gustavo Deco

**Affiliations:** 1 Department of Technology, Universitas Pompeu Fabra, Barcelona, Spain; 2 Department of Experimental Psychology, University of Oxford, Oxford, United Kindgom; 3 Institució Catalana de Recerca i Estudis Avançats, Barcelona, Spain; University College London, United Kingdom

## Abstract

We propose a top-down approach to the symptoms of schizophrenia based on a statistical dynamical framework. We show that a reduced depth in the basins of attraction of cortical attractor states destabilizes the activity at the network level due to the constant statistical fluctuations caused by the stochastic spiking of neurons. In integrate-and-fire network simulations, a decrease in the NMDA receptor conductances, which reduces the depth of the attractor basins, decreases the stability of short-term memory states and increases distractibility. The cognitive symptoms of schizophrenia such as distractibility, working memory deficits, or poor attention could be caused by this instability of attractor states in prefrontal cortical networks. Lower firing rates are also produced, and in the orbitofrontal and anterior cingulate cortex could account for the negative symptoms, including a reduction of emotions. Decreasing the GABA as well as the NMDA conductances produces not only switches between the attractor states, but also jumps from spontaneous activity into one of the attractors. We relate this to the positive symptoms of schizophrenia, including delusions, paranoia, and hallucinations, which may arise because the basins of attraction are shallow and there is instability in temporal lobe semantic memory networks, leading thoughts to move too freely round the attractor energy landscape.

## Author Summary

One of the hallmarks of schizophrenia is the complexity and heterogeneity of the illness. We propose that part of the reason for the inconsistent symptoms may be a reduced signal-to-noise ratio and increased statistical fluctuations in different cortical brain networks. The novelty of the approach described here is that instead of basing our hypothesis purely on biological mechanisms, we develop a top-down approach based on the different types of symptoms and relate them to instabilities in attractor neural networks. Schizophrenia is characterized by cognitive, negative, and positive symptoms. We propose which characteristic effects in a dynamical system could cause these symptoms, and investigate our hypothesis in a computational model. We implement an integrate-and-fire network model and focus on the alterations of synaptic channels activated via NMDA and GABA receptors. We found that a decrease in the NMDA receptor conductance could contribute to both the cognitive and negative symptoms by reducing the neuronal firing rates and the stability of the attractor states. A reduction of both NMDA and GABA conductance causes an instability of the attractor states related to the positive symptoms. Overall, we provide a framework to discuss schizophrenia in a dynamical system framework.

## Introduction

Schizophrenia is a major mental illness, which has a great impact on patients and their environment. One of the difficulties in proposing models for schizophrenia is the complexity and heterogeneity of the illness. We propose that part of the reason for the inconsistent symptoms may be a reduced signal-to-noise ratio and increased statistical fluctuations in different cortical brain networks. The novelty of the approach described here is that instead of basing our hypothesis purely on biological mechanisms, we develop a top-down approach based on the different types of symptoms and relate them to instabilities in attractor neural networks [1].

The main assumption of our hypothesis is that attractor dynamics are important in cognitive processes [2]. Our hypothesis is based on the concept of attractor dynamics in a network of interconnected neurons that in their associatively modified synaptic connections store a set of patterns, which could be memories, perceptual representations, or thoughts [3–5]. The attractor states are important in cognitive processes such as short-term memory, attention, and action selection [6]. The network may be in a state of spontaneous activity, or one set of neurons may have a high firing rate, each set representing a different memory state, normally recalled in response to a retrieval stimulus. Each of the states is an attractor in the sense that retrieval stimuli cause the network to fall into the closest attractor state, and thus to recall a complete memory in response to a partial or incorrect cue. Each attractor state can produce stable and continuing or persistent firing of the relevant neurons. The concept of an energy landscape [4] is that each pattern has a basin of attraction, and each is stable if the basins are far apart and also if each basin is deep, which is caused, for example, by high firing rates and strong synaptic connections between the neurons representing each pattern, which together make the attractor state resistant to distraction by a different stimulus. The spontaneous firing state, before a retrieval cue is applied, should also be stable. Noise in the network caused by statistical fluctuations in the stochastic spiking of different neurons can contribute to making the network transition from one state to another; we take this into account by performing integrate-and-fire simulations with spiking activity, and relate this to the concept of an altered signal-to-noise ratio in schizophrenia [7–9].

Schizophrenia is characterized by three main types of symptom: cognitive dysfunction, negative symptoms, and positive symptoms [10–12]. We consider how the basic characteristics of these three categories might be produced in a neurodynamical system, as follows. Dysfunction of working memory, the core of the cognitive symptoms, may be related to instabilities of persistent attractor states [13,14], which we show can be produced by reduced firing rates in attractor networks in brain regions such as the prefrontal cortex. The negative symptoms such as flattening of affect or reduction of emotions may be caused by a consistent reduction in firing rates of neurons in regions associated with emotion, such as the orbitofrontal cortex [1]. These hypotheses are supported by the frequently observed hypofrontality, a reduced activity in frontal brain regions in patients with schizophrenia during cognitive tasks [15–17]. The positive symptoms are characterized by phenomenologically overactive perceptions or thoughts such as hallucinations or delusions, which are reflected, for example, by higher activity in the temporal lobes [17,18]. We relate this category of symptoms to a spontaneous appearance of activity in attractor networks in the brain and more generally to instability of both the spontaneous and persistent attractor states. We do not try to account for every detail of schizophrenic symptoms, which are diverse and vary among patients, but instead show how these three main categories of symptoms could be related to changes in the stability of dynamical attractor systems in the brain, and how the changes in the stability might be produced by changes at the level of the currents being passed through receptor-activated ion channels at synapses. There are specific symptoms such as aberrant eye movements that cannot be accounted for by this general scheme.

In particular, we were interested in how these symptoms are related. Negative and cognitive symptoms typically precede the first psychotic episode [19,20]. Positive symptoms can be treated in most cases with neuroleptics, whereas negative and cognitive symptoms persist, at least for typical neuroleptics. Can a mapping onto a dynamical system help to understand these relations? After proposing a dynamical systems hypothesis for the different symptoms of schizophrenia, we study a standard neural network model [21] of cortical dynamics specifically in relation to our hypothesis. We were especially interested in how excitation and inhibition implemented by NMDA and GABA synapses affect the network dynamics. Alterations in the efficacies of the NMDA and GABA channels have been identified in the pathology of schizophrenia [22,23], and transmitters such as dopamine influence the currents in these receptor-activated channels [24]. Do NMDA and GABA currents have antagonistic effects, or do they have a special role in the network dynamics? How could this be related to our hypothesis of schizophrenia? Building upon the current body of neural network research, we specifically conduct neural network simulations to substantiate our dynamical systems hypothesis of schizophrenia. While focussing on NMDA and GABA synapses in the Results, in the Discussion we consider how altered transmission at D1 and D2 receptors by modulating NMDA and GABA conductances could not only influence working memory, which has been investigated previously [21,25–27], but could in particular influence the different symptoms of schizophrenia.

## Results

### Dynamical Systems Hypothesis

We relate the three types of symptoms of schizophrenia to the dynamical systems attractor framework described at the end of the Introduction as follows [1].

The cognitive symptoms of schizophrenia include distractibility, poor attention, and the dysexecutive syndrome [10–12,28]. The core of the cognitive symptoms is a working memory deficit in which there is a difficulty in maintaining items in short-term memory [29,30], which could directly or indirectly account for a wide range of the cognitive symptoms. We propose that these symptoms may be related to instabilities of persistent states in attractor neural networks, consistent with the body of theoretical research on network models of working memory [13]. The neurons are firing at a lower rate, leading to shallower basins of attraction of the persistent states, and thus a difficulty in maintaining a stable short-term memory, normally the source of the bias in biased competition models of attention [5,6]. The shallower basins of attraction would thus result in working memory deficits, poor attention, distractibility, and problems with executive function and action selection [6,31].

The negative symptoms refer to the flattening of affect and a reduction in emotion. Behavioural indicators are blunted affect, emotional and passive withdrawal, poor rapport, lack of spontaneity, motor retardation, and disturbance of volition [10–12]. We propose that these symptoms are related to decreases in firing rates in the orbitofrontal cortex and/or anterior cingulate cortex [1], where neuronal firing rates and activations in functional MRI investigations are correlated with reward value and pleasure [1,32]. Consistent with this, imaging studies have identified a relationship between negative symptoms and prefrontal hypometabolism (i.e., a reduced activation of frontal areas [33,34]).

The positive symptoms of schizophrenia include bizarre (psychotic) trains of thought, hallucinations, and (paranoid) delusions [10–12]. We propose that these symptoms are related to shallow basins of attraction of both the spontaneous and persistent states in the temporal lobe semantic memory networks and to the statistical fluctuations caused by the probabilistic spiking of the neurons. This could result in activations arising spontaneously and thoughts moving too freely around the energy landscape, loosely from thought to weakly associated thought, leading to bizarre thoughts and associations that may eventually over time be associated together in semantic memory to lead to false beliefs and delusions. Consistent with this, neuroimaging studies suggest higher activation, especially in areas of the temporal lobe [17,18,35].

To further investigate our hypothesis, we use an attractor network, as this is likely to be implemented in many parts of the cerebral cortex by the recurrent collateral connections between pyramidal cells, and has short-term memory properties with basins of attraction that allow systematic investigation of stability and distractibility. The particular neural network implementation we adopt includes channels activated by AMPA, NMDA, and GABA_A_ receptors, and allows not only the spiking activity to be simulated, but also a consistent mean-field approach to be used [21].

### The Depth of the Basins of Attraction: Flow Analysis

First, we introduce an analytical approach to the concepts of how changes in transmitters could affect the depth of the basins of attraction in networks in ways that may be related to the symptoms of schizophrenia.

**Figure 1:**
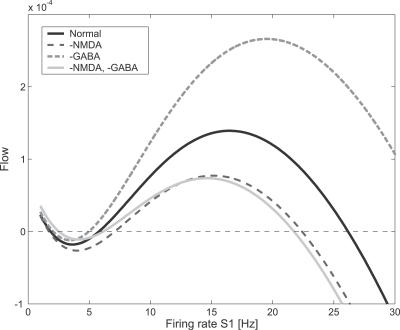
Flow of a Single-Attractor Network We assess the flow of an attractor network with one selective pool using the mean-field analysis for different modifications of synaptic conductances (see Methods section). The flow represents the force which drives the system toward one of the stable attractors. The stable/unstable attractor states are at crossings with the flow = 0 value on the flow axis with a negative/positive derivative, respectively. A modulation of the synapses labelled as (−NMDA) and (−GABA) corresponds to a reduction of 4.5% and 9% of the efficacies, respectively. A pool cohesion of *w_+_* = 1.6 and a selective pool size of 80 neurons were used for the simulations.

The depth of the basins of attraction can be assessed by calculating the flow toward the attractors using the mean-field reduction (see Methods and [36]). The flow is the force that draws the dynamical system to one of the attractors. Figure 1 shows the flow between the spontaneous and persistent state in a network featuring one selective pool. The curve for (Normal) shows the flow for the reference baseline condition. The crossing of the curve and the 0-flow axis represent the fixed points, which are either stable (negative derivative) or unstable (positive derivative). We use this line as a reference to assess the relative depth of the energy landscape. The system flows either into a spontaneous firing state (of approximately 2 Hz), or a high-firing attractor state.

A reduction of NMDA (−NMDA) shows a stronger flow than the unchanged condition at low firing rates toward the spontaneous attractor (at about 2 Hz). The absolute values of the function are higher compared with the normal condition until the first unstable fixed point (at around 6–7 Hz). The basin of attraction toward the persistent attractor at high firing rates yields the reverse picture. Here, the −NMDA curve is clearly below the unchanged condition, and the flow toward the attractor is smaller. Overall, the basin of attraction is deeper for the spontaneous state and shallower for the persistent state compared with the unchanged condition. This pattern fits with the cognitive symptoms of schizophrenia as proposed in our hypothesis. Also, note that the firing rate of the persistent fixed point is reduced in the −NMDA condition (crossing with the flow = 0-axis), which is consistent with the hypothesis for the negative symptoms.

A reduction of the GABA conductance (−GABA) yields the opposite pattern to that in the reduced NMDA condition. Here, the basin of attraction of the persistent state is deeper. This is not a condition that we suggest is related to the symptoms of schizophrenia.

However, in the condition in which both the NMDA and the GABA conductances are reduced (−NMDA, −GABA), the persistent-state basin of attraction is shallower, and the spontaneous-state basin is a little shallower. This condition corresponds to the proposed landscape for the positive symptoms as considered above. In particular, in the −NMDA, −GABA condition, the system would be less stable in the persistent state, tending to move to another attractor easily, and less stable in the spontaneous state, also tending to move too readily into an attractor from spontaneous activity.

Overall, the flow analysis suggests that both the cognitive and negative symptoms could be related to a decrease in the NMDA conductances. This is consistent with the fact that these two symptoms usually appear together. The flow analysis suggests that the positive symptoms are related to a reduction in both NMDA and GABA. Thus, the transition from the cognitive and negative symptoms to the positive, psychotic symptoms might be caused by an additional decrease in the GABA conductance. It is notable that excitation and inhibition do not cancel each other out as assumed by many models, but have distinct influences on the network dynamics.

### Concept of Stability in Network Simulations

The flow analysis provides insight into how the depth of the basins of attraction and the firing rates are influenced by changes in the conductivities of the channels activated via NMDA and GABA receptors. However, the overall stability of the different attractors is affected not only by the depth of the basins of attraction, but also by the breadth and distance apart of the basins and by the statistical noise generated by the randomness of the spiking of the different neurons. These statistical fluctuations play a role in the way in which the system moves from one state to another, for these statistical fluctuations can cause hills in the energy landscape to be crossed stochastically. Since the mean-field analyses do not take these properties into account, we investigate the system further using large-scale integrate-and-fire network simulations and measuring the statistics of the network behavior.

**Figure 2:**
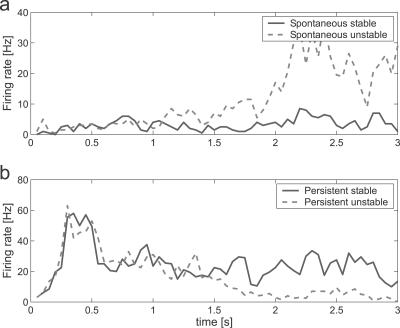
Example Trials of the Integrate-and-Fire Attractor Network Simulations (A) Trials in which no recall stimulus was applied to S1, and spontaneous firing continued until the end of the trial in the “spontaneous” simulation condition. However, on one of the trials illustrated, the firing increased as the network climbed into one of the attractors because of the statistical fluctuations caused by the spiking dynamics. (B) Trials in which a recall stimulus was applied to S1 at 0–500 ms, and continuing firing normally occurred until the end of the trial in the “persistent” simulation condition. However, on one of the trials illustrated, the firing decreased as the network fell out of the attractor because of the statistical fluctuations caused by the spiking dynamics. In these simulations, the network parameter is *w_+_* = 2.1, and the modulation of the synapses is −NMDA and −GABA. This corresponds to a reduction of 4.5% and 9% in the currents produced through these synapses, respectively. The average firing rate of all the neurons in the S1 pool is shown.

To clarify the concept of stability, we show examples of trials of spontaneous and persistent simulations in which the statistical fluctuations have different effects on the temporal dynamics. Figure 2 shows the possibilities, as follows.

In the spontaneous-state simulations, no cue is applied, and we are interested in whether the network remains stably in the spontaneous firing state, or whether it is unstable and, on some trials due to statistical fluctuations, enters one of the attractors, thus falsely retrieving a memory. Figure 2A shows an example of a trial on which the network correctly stays in the low spontaneous firing rate regime, and another trial (labelled spontaneous unstable) in which statistical spiking-related fluctuations in the network cause it to enter a high-activity state, moving into one of the attractors even without a stimulus.

In the persistent-state simulations, a strong excitatory input is given to the S1 neuronal population between 0 and 500 ms (see Analysis section). Two such trials are shown in Figure 2B. In one, the S1 neurons (correctly) keep firing at approximately 30 Hz after the retrieval cue is removed at 500 ms. However, due to statistical fluctuations in the network related to the spiking activity, on the trial labelled persistent unstable, the high firing rate in the attractor for S1 was not stable, and the firing decreased back toward the spontaneous level, in the example shown starting after 1.5 s. This trial illustrates the failure to maintain a stable short-term memory state, even when no distractor is applied.

In Figure 2, the transitions to the incorrect activity states are caused by statistical fluctuations in the spiking activity of the integrate-and-fire neurons and the depth of the basins of attraction, which has been reduced in the simulations shown by reducing both the NMDA and the GABA currents, as indicated in the caption. We hypothesize that such instabilities are related to the symptoms of schizophrenia. We note that there are two sources of noise in the spiking networks that cause the statistical fluctuations: the randomly arriving external Poisson spike trains, and the statistical fluctuations caused by the spiking of the neurons in the finite sized network. The magnitude of these fluctuations increases as the number of neurons in the network becomes smaller [37].

For our investigations, we selected *w*_+_ = 2.1, which with the default values of the NMDA and GABA conductances yielded stable dynamics; that is, a stable spontaneous state if no retrieval cue was applied, and a stable state of persistent firing after a retrieval cue had been applied and removed. To investigate the effects of changes (modulations) in the NMDA and GABA conductances, we chose for demonstration purposes a reduction of 4.5% and 9%, respectively, as these could cause instabilities, as illustrated in Figure 2. However, the exact values are not crucial to observe the effects described. The magnitudes of these reductions are smaller than those that can be produced experimentally [24,26]. A strength of our approach is that we show that even quite small reductions in the synaptic currents can alter the global behaviour of the network, e.g., the stability of its attractors.

### Stability

**Figure 3:**
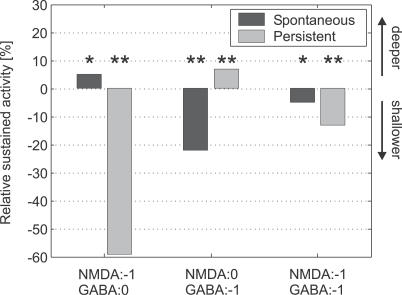
Stability of the Spontaneous and Persistent States We assessed how often in 100 trials the average activity during the last second (2–3 s) stayed above 10 Hz. The value shows how often it stayed more in the respective state than in the unmodulated reference state. A negative percentage means that the system was less stable than in the reference state. A modulation of the synapses shown as −NMDA and −GABA corresponds to a reduction of 4.5% and 9% in their efficacies, respectively. We assessed with the binomial distribution the statistical significance of the effects observed; **p < 0.01 relative to the reference state, and *p < 0.02.

We assessed how the stability of both the spontaneous and persistent states changes when NMDA and GABA efficacies are modulated. Specifically, we ran multiple-trial integrate-and-fire network simulations and counted how often the system maintained the spontaneous or persistent state, assessed by the firing rate in the last second of the simulation (2–3 s) of each 3-s trial. Figure 3 shows the stability of the spontaneous and persistent attractors relative to the unmodulated reference state (Normal). A negative percentage means that the system was less stable than in the unmodulated state.

A reduction of the NMDA conductance (−NMDA) reduces the stability of the persistent state drastically, while slightly increasing the stability of the spontaneous state (see Figure 3). We hypothesized that this type of change might be related to the cognitive symptoms, since it shows a reduced stability of the working memory properties. A reduction of GABA shows the opposite pattern: a slight reduction in the stability of the spontaneous state, and an increased stability of the persistent (i.e., attractor) state (see Figure 3).

When both NMDA and GABA are reduced, one might think that these two counterbalancing effects (excitatory and inhibitory) would either cancel each other out or yield a tradeoff between the stability of the spontaneous and persistent states. However, this is not the case. The stability of both the spontaneous state and the persistent state is reduced (see Figure 3). We relate this pattern to the positive symptoms of schizophrenia, in which both the spontaneous and attractor states are shallow, and the system merely jumps by the influence of statistical fluctuations between the different (spontaneous and attractor) states.

**Figure 4:**
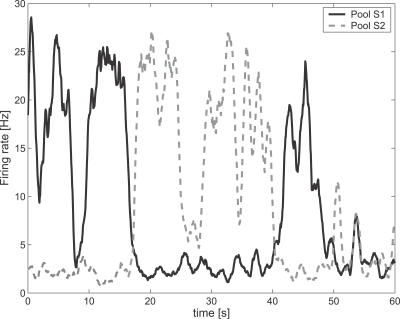
Wandering between Attractor States We simulated a single long trial (60 s) in the spontaneous test condition for the synaptic modulation (−NMDA, −GABA). The two curves show the activity of the two selective pools over time smoothed with a 1-s sliding averaging window. The activity moves noisily between the attractor for the spontaneous state and the two persistent states S1 and S2 by virtue of statistical fluctuations caused by the randomness of the spiking activity.

To investigate more directly the wandering between spontaneous and several different persistent attractor states, we simulated the condition with decreased NMDA and GABA conductances over a long time period in which no cue stimulus input was given. Figure 4 shows the firing rates of the two selective pools S1 and S2. The high activity switches between the two attractors due to the influence of fluctuations, which corresponds to spontaneous wandering in a shallow energy landscape, corresponding, for example, to sudden jumps between unrelated cognitive processes. These results are consistent with the flow analysis and demonstrate that the changes in the attractor landscape influence the behavior at the stochastic level.

### Distractibility

**Figure 5:**
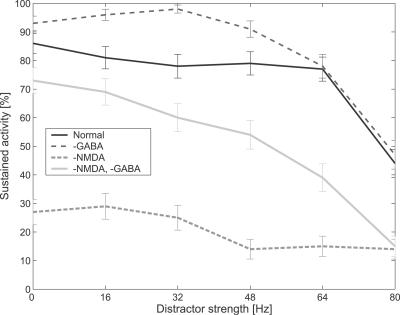
Stability and Distractibility as a Function of the Distractor Strength and the Synaptic Efficacies We assessed how often in 100 trials the average activity during the last second (2–3 s) stayed above 10 Hz in the S1 attractor. The modulation of the synapses NMDA: −1 and GABA: −1 corresponds to a reduction of 4.5% and 9%, respectively. The strength of the distractor stimulus applied to S2 is an increase in firing rate above the 2.4-kHz background activity, which is distributed among 800 synapses per neuron. The lower the sustained activity in S1, the higher is the distractibility. The standard deviations were approximated with the binomial distribution.

As distractibility is directly related to the symptoms of schizophrenia, we ran simulations specifically to assess this property using persistent and distractor simulations (see Analysis section). A distractor strength of 0 Hz corresponds to the persistent condition described in the preceding section (Stability). Figure 5 shows the stability and distractibility for reductions of NMDA and GABA currents. The reference state is labelled “Normal.” In this state, pool S1 continued to maintain its attractor firing without any distractor (distractor strength = 0 Hz) throughout the delay period on almost 90% of the trials. In both conditions that reduce the NMDA current (labelled −NMDA), the network was less and less able to maintain the S1 attractor firing as the distractor stimulus strength was increased through the range of 0–80 Hz. The stability of the persistent state was reduced, and the distractibility was also increased, as shown by the fact that increasing distractor currents applied to S2 could move the attractor away from S1. The implication, therefore, is that a reduction of the NMDA currents could cause the cognitive symptoms of schizophrenia by making short-term memory networks less stable and more distractible, thereby reducing the ability to maintain attention. Reducing only the GABA currents (−GABA) reduces the distractibility for low distractor strengths and coincides with the reference (Normal) condition at high values of the distractor strengths.

### Signal-to-Noise Ratio

**Figure 6:**
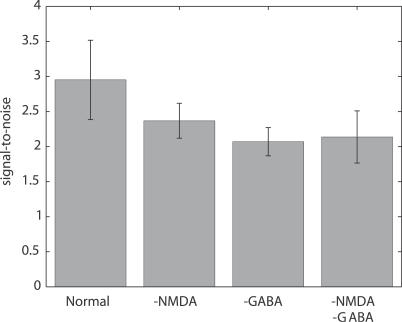
Signal-to-Noise Ratio of a Measure Related to the Blood Oxygenation Level–Dependent Signal We computed the mean and standard deviation of averages of the synaptic currents of the selective pool over the whole simulation period of a persistent condition simulation. The mean of the spontaneous baseline condition was subtracted. We conducted 1,000 simulated trials. The signal-to-noise ratio is calculated by division of the mean synaptic current by the standard deviation measured using 1,000 trials. The error bars indicate an estimate of the standard deviation measured over 20 epochs containing 50 trials each. A modulation of the synapses shown as −NMDA and −GABA corresponds to a reduction of 4.5% and 9% in their conductances, respectively.

We further investigated the signal-to-noise ratio in relation to the changes in synaptic conductances. The signal-to-noise ratio denotes the level of a signal relative to the level of background noise. In an attractor network, a high signal-to-noise ratio indicates that the network will maintain the attractor stably, as it will be unlikely to be disrupted by spiking-related statistical fluctuations that are the source of the noise in the network. Figure 6 shows the signal-to-noise ratio of a measure related to the functional MRI blood oxygenation level–dependent signal. (This measure described in the caption to Figure 6 and below was used because the experimental data with which we wish to compare the simulation results use functional MRI measures [7–9]. The index we used of the activity of the network was the total synaptic current of selective pool 1 averaged over the whole simulation time of 3 s to take the temporal filtering properties of the blood oxygenation level–dependent signal into account, given the typical time course which lasts for several seconds of the haemodynamic response function [38]. Further, we subtracted the averages of the spontaneous trial simulations that represent the baseline activity from the persistent trial simulation values. The signal-to-noise ratio was calculated from the mean of this index across trials divided by the standard deviation of the index, both measured using 1,000 simulation trials. If the network sometimes had high activity, and sometimes low, then the signal-to-noise measure gave a low value. If the network reliably stayed in the high persistent firing states, then the signal-to-noise ratio measure was high.) As shown in Figure 6, we found that in all the cases in which the NMDA or the GABA conductance, or both, are reduced, the signal-to-noise ratio, computed by the mean divided by the standard deviation, is also reduced. This relates to recent experimental observations which show a decreased signal-to-noise ratio in schizophrenic patients [7–9]. Here, we directly relate a decrease in the signal-to-noise ratio to changes (in this case, decreases) in receptor-activated synaptic channel conductances. Given these results, it would be of interest in future studies to model the exact paradigm used by Winterer et al. [8].

## Discussion

**Figure 7:**
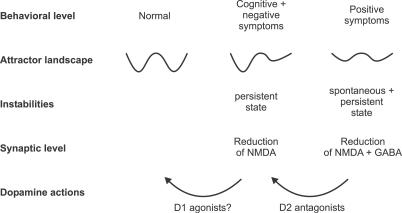
Summary of the Attractor Hypothesis of Schizophrenic Symptoms and the Simulation Results The first basin (from the left) in each energy landscape is the spontaneous state, and the second basin is the persistent attractor state. The vertical axis of each landscape is the energy potential. See text for simulation results.

We have proposed a hypothesis that relates the cognitive, negative, and positive symptoms of schizophrenia [10–12] to the depth of basins of attraction and to the stability properties of attractor networks caused by statistical fluctuations of spiking neurons. This assumes that some cognitive processes can be understood as dynamical attractor systems, which is an established hypothesis in areas such as working memory, but has also been used in many other areas [2,5]. Our approach applies this concept to mental illnesses [39]. Due to the diversity of schizophrenic symptoms, our general hypothesis is meant to serve as a heuristic of how the different kinds of symptoms might arise and are related. We investigated the hypothesis empirically in a computational attractor framework to capture an important aspect of cortical functionality. Figure 7 summarizes our hypothesis and its relation to the investigations of an attractor neural network.

The middle column in Figure 7 shows the overview for the cognitive and negative symptoms. The core of the cognitive symptoms is a failure of working memory and attentional mechanisms. Working memory activity is related to the ongoing (i.e., persistent) firing of neurons during the delay period of cognitive tasks [29,30]. This could be implemented by associatively modifiable synapses between the recurrent collateral synapses of cortical pyramidal cells [13,14,40,41]. We propose that the cognitive symptoms of schizophrenia could arise because the basins of attraction of the persistent states in the prefrontal cortex become too shallow. This leads in combination with the statistical fluctuations due to randomness of the spiking activity to either a fallout of an active working memory state or to a shift to a different attractor state, leading to a failure to maintain attention and thereby impairing executive function. The hypofrontality in schizophrenia, that is, less activation in frontal brain regions during working memory tasks [15,42], is in line with our hypothesis, since the firing rates of the persistent state are lower in the reduced NMDA condition (Figure 1), and the system spends on average less time in the persistent state, since it is less stable than in the normal condition (Figure 3). In addition, a reduced signal-to-noise ratio as shown in our simulations (Figure 6) has also been identified in imaging studies [7–9]. Our simulations suggest that a reduction in NMDA conductance at the synaptic level (see Figure 7) can account for this phenomenon. This is in line with previous work on the stability of working memory networks [14,27,43].

A reduction of the NMDA conductance also results in a reduction of the firing rates of the neurons in the persistent state (see Figure 1 and [21]). We relate this, following [1], to the negative symptoms, which include flattening of affect, a reduction in emotion, emotional and social withdrawal, poor rapport, passive withdrawal, lack of spontaneity, motor retardation, apathy, and disturbance of motivation. These symptoms are related to decreases in activity in the orbitofrontal cortex and/or anterior cingulate cortex [33,34], both of which are implicated in emotion [1,32,44]. The emotional states represented in the orbitofrontal cortex and anterior cingulate cortex include states elicited both by rewards and punishers. Our hypothesis is that both would be reduced by the mechanism described. Correspondingly, motivation would be reduced in the same way, in that motivation is a state in which we work to obtain goals (rewards) or avoid punishers [1].

Both the negative and cognitive symptoms thus could be caused by a reduction of the NMDA conductance in attractor networks. The proposed mechanism links the cognitive and negative symptoms of schizophrenia in an attractor framework and is consistent with a close relation between the cognitive and negative symptoms: blockade of NMDA receptors by dissociative anesthetics such as ketamine produces in healthy subjects schizophrenic symptoms, including both negative and cognitive impairments [45,46]; agents that enhance NMDA receptor function reduce the negative symptoms and improve the cognitive abilities of schizophrenic patients [47]; and the cognitive and negative symptoms occur early in the illness and precede the first episode of positive symptoms [12,19,20]. Consistent with this hypothesized role of a reduction in NMDA conductances being involved in schizophrenia, postmortem studies of schizophrenia have identified abnormalities in glutamate receptor density in regions such as the prefrontal cortex, thalamus, and the temporal lobe [22,47], brain areas that are active during the performance of cognitive tasks.

The dopamine D1 receptor has been shown to modulate the performance of working memory tasks [30,48–50]. An increase in D1 receptor activation has been shown to increase the NMDA current [24,26], and modeling studies have shown that this increase is related to the stability of working memory states [21,25,27]. Imaging data also support the importance of the D1 receptor in schizophrenia [51,52]. We therefore suggest that an increased activation of D1 receptors might alleviate both the cognitive and the negative symptoms of schizophrenia [53,54] by increasing NMDA receptor-mediated synaptic currents (Figure 7). Atypical neuroleptics might use this mechanism by not only blocking D2 receptors, but also by increasing the presynaptic release of dopamine, which in turn would increase the activation of the extrasynaptic D1 receptors [48,55].

Taken together, we suggest that the cognitive and negative symptoms could be caused by the same synaptic mechanism, namely a reduction in the NMDA conductance, which reduces the stability and increases the distractibility of the persistent attractors, and reduces the activity (firing rates) of neurons (Figure 7, middle column). The reduced depth of the basins of attraction can be understood in the following way. Hopfield [4] showed that the recall state in an attractor network can be thought of as the local minimum in an energy landscape, where the energy would be defined as

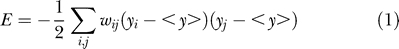
where *y_i_* and *y_j_* are the firing rates of the *i*th and *j*th neurons in the network, which are connected by synaptic weight *w_ij_*. In general, neuronal systems do not admit an energy function. Nevertheless, we can assume an effective energy function: in fact, the flow picture shown in Figure 1 resulting from the mean-field reduction associated with the spiking network analyzed here can be viewed as an indirect description of an underlying effective energy function. From this equation, it follows that the depth of a basin of attraction is deeper if the firing rates are higher and if the synaptic strengths that couple the neurons that are part of the same attractor are strong. (The negative sign results in a low energy, and thus a stable state, if the firing rates of the neurons in the same attractor and their synaptic coupling weights are high.) If we reduce the NMDA receptor–activated channel conductances, then the depth of the basins of attraction will be reduced both because the firing rates are reduced by reducing excitatory inputs to the neurons, and because the synaptic coupling weights are effectively reduced because the synapses can pass only reduced currents.

The positive symptoms (Figure 7, right column) of schizophrenia include delusions, hallucinations, thought disorder, and bizarre behavior. Examples of delusions are beliefs that others are trying to harm the person, impressions that others control the person's thoughts, and delusions of grandeur. Hallucinations are perceptual experiences that are not shared by others and are frequently auditory, but can affect any sensory modality. These symptoms may be related to activity in the temporal lobes [11,12,56]. The attractor framework approach taken in this paper hypothesizes that the basins of attraction of both spontaneous and persistent states are shallow (Figure 7). Due to the shallowness of the spontaneous state, the system can jump spontaneously up to a high activity state, causing hallucinations to arise and leading to bizarre thoughts and associations. This might be the cause for the higher activations in temporal lobe areas that are identified in imaging experiments [17,18].

We relate the positive symptoms to not only a reduction in NMDA conductance, but also to a reduction in GABA conductance. This is consistent with the fact that the positive symptoms usually follow the cognitive and negative symptoms and represent a qualitative worsening of the illness [12]. Alterations in GABA receptors have been identified in schizophrenia [23,57].

D2 receptor antagonism remains a main target for antipsychotics [58,59]. Dopamine receptor D2 antagonists mainly alleviate the positive symptoms of schizophrenia, whereas the cognitive and negative symptoms persist, especially for the typical neuroleptics [12]. Together with the simulations, our hypothesis suggests that an increase in the GABA current in the state corresponding to the positive symptoms (−NMDA, −GABA) might have the same effect as D2 antagonists. The therapeutic effect of D2 antagonists might thus be caused by an increase in GABA currents. Indeed, it has been found that D2 receptors decrease the efficacy of the GABA system [60,61]. (For example, the application of D2 antagonists prevented a decrease in evoked inhibitory postsynaptic current amplitude produced by dopamine [60].) Thus, D2 antagonists would, in a hypersensitive D2 receptor state [62,63], increase GABA inhibition in the network, and we suggest could increase the stability of attractor networks involved in the positive symptoms of schizophrenia, and thus ameliorate the positive symptoms. Since the concentration of dopamine in the cortex depends on cortical–subcortical interactions [64], the causes of the described changes could also result from subcortical deficits. A detailed analysis of these feedback loops would require specific modeling.

Earlier accounts of the relation of dopamine and schizophrenia in the cortex [24,60] have suggested two distinct states of dopamine modulation. One is a D2 receptor–dominated state in which there is weak gating and in which information can easily affect network activity. The other is a D1 receptor–dominated state in which network activity is stable and maintained. We have proposed a more detailed account for stability and discussed this separately for the spontaneous and persistent attractor states. This allows us to account for the dichotomy between the cognitive/negative and positive symptoms. We emphasize that in biophysically realistic network simulations, excitation and inhibition are not merely antagonistic but implement different functions in the network dynamics. Thereby, our modeling approach provides a missing link between the symptoms of schizophrenia and network models of working memory and dopamine [21,25,27]. This approach is not meant to provide a detailed discussion of specific symptoms of schizophrenia. Further research would be needed to relate specific symptoms to the overall scheme presented here in this paper.

We concentrated in our study on basic effects in local cortical dynamics based on biophysically realistic spiking networks. One can extend the model to study the interaction between large-scale networks involving more than one location in the cortex and/or subcortical regions. This increases the dimensionality of the system and adds to the issues of stability [65,66]. Processes such as bubbling transitions and chaotic attractors also add to the notion of stability [67,68]. For high-dimensional systems, Kaneko uses the concept of a Milnor attractor [69], which offers a way to formally describe the stability of an attractor. This approach measures the stability of an attractor against perturbations by introducing the concept of “return probability.” By sampling over random perturbation and orbit positions, the return probability is defined as the fraction of trials in which the system returns back to the original point. This measures the strength of the attractor. In our work, we wanted to stay close to paradigms of working memory and address mechanisms involved in cognitive processes, attention, and distractibility. Consequently, our quantitative measures of stability are intrinsically related to these paradigms and therefore use concepts such as the escape probability instead of the return probability. Indeed, we introduced these measures in order to relate the quantitative concept of stability more directly with the cognitive symptoms. Nevertheless, in detailed investigations of specific symptoms of schizophrenia, the concepts mentioned above should be kept in mind, as they might play a role in neural dynamics.

Cohen and collaborators focused in a series of computational and experimental studies on the cognitive symptoms of schizophrenia. Their connectionist models try to account for specific experimental data on context-processing deficits using working memory, gating, and attentional selection as mechanisms [70–72]. The context-processing hypothesis is compatible with the hypothesis of working memory deficits in schizophrenia, as context processing relies on stable working memory: a deficit in working memory would consequently lead to a deficit in context processing. Our model is conceptually at another level, featuring biophysically plausible single-neuron properties and specific synaptic currents. It is not intended to account for specific experimental data, but provides insights at a more generic and also biological level. In this sense, the two models complement each other.

We discussed a possible cause for the proposed alterations of the attractor landscape related to schizophrenia, namely changes in NMDA and GABA conductance, as these are directly related to schizophrenia [22,23]. We did not investigate changes in AMPA conductance. In this particular model, the contribution of the AMPA current is relatively small [21]. A more detailed investigation could also include AMPA conductance, especially because it is known to be influenced by NMDA synaptic plasticity [73]. Indeed, if reduced NMDA currents led in turn by synaptic plasticity to reduced AMPA currents, this would amplify the effects we describe. The proposed alterations in the attractor landscape could have a variety of causes at the neurobiological level: abnormalities in glutamate and GABA receptors and signaling, modulations in synaptic plasticity, aberrant dopamine signaling, reduced neuropil, genetic mechanisms, and brain volume reduction [12,30,74,75]. Besides cortical mechanisms, cortical–subcortical dynamics could also cause the proposed alterations in the cortical attractor landscape, for example, via neuromodulatory influences such as dopamine or serotonin or cortical–subcortical feedback loops [64,76]. Our general hypothesis regarding the attractor landscape is meant to describe the aberrant dynamics in cortical regions that could be caused by several pathways. Future work could analyze further how changes of different factors such as regional differences, subcortical–cortical networks, or even more detailed neural and synaptic models might influence the stability of the type of neurodynamical system described here. We envision that our hypothesis could serve as a useful guideline for further theoretical work.

## Methods

### Attractor framework.

Our aim is to investigate stability and distractibility in a biophysically realistic attractor framework so that the properties of receptors, synaptic currents, and the statistical effects related to the probabilistic spiking of the neurons can be part of the model. We use a minimal architecture, a single-attractor or autoassociation network [3–5,41,77]. We chose a recurrent (attractor) integrate-and-fire network model which includes synaptic channels for AMPA, NMDA, and GABA_A_ receptors [21]. These synaptic receptors are important, since there is evidence that alterations in synaptic currents are related to the symptoms of schizophrenia, and many of the drugs used to treat schizophrenia act on these receptor-mediated currents, either directly or indirectly [22, 23].

Both excitatory and inhibitory neurons are represented by a leaky integrate-and-fire model [78]. The basic state variable of a single-model neuron is the membrane potential. It decays in time when the neurons receive no synaptic input down to a resting potential. When synaptic input causes the membrane potential to reach a threshold, a spike is emitted and the neuron is set to the reset potential at which it is kept for the refractory period. The emitted action potential is propagated to the other neurons in the network. The excitatory neurons transmit their action potentials via the glutamatergic receptors AMPA and NMDA, which are both modeled by their effect in producing exponentially decaying currents in the postsynaptic neuron. The rise time of the AMPA current is neglected because it is typically very short. The NMDA channel is modeled with an alpha function, including both a rise and a decay term. In addition, the synaptic function of the NMDA current includes a voltage dependence controlled by the extracellular magnesium concentration [79]. The inhibitory postsynaptic potential is mediated by a GABA_A_ receptor model and is described by a decay term.

The single-attractor network contains 400 excitatory and 100 inhibitory neurons, which is consistent with the observed proportions of pyramidal cells and interneurons in the cerebral cortex [80,81]. The connection strengths are adjusted using mean-field analysis [21] so that the excitatory and inhibitory neurons exhibit a spontaneous activity of 3 Hz and 9 Hz, respectively [82,83]. The recurrent excitation mediated by the AMPA and NMDA receptors is dominated by the NMDA current to avoid instabilities during the delay periods [84].

**Figure 8:**
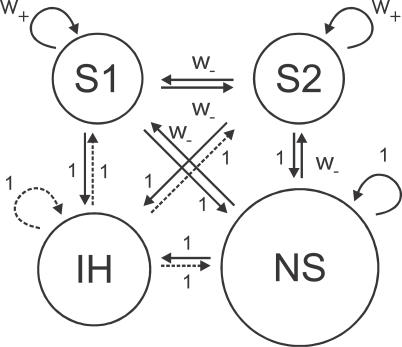
The Attractor Network Model The excitatory neurons are divided into two selective pools, S1 and S2 (with 40 neurons each), with strong intrapool connection strengths *w_+_* and one nonselective pool (NS) (with 320 neurons). The other connection strengths are 1 or weak *w*_−_. The network contains 500 neurons, of which 400 are in the excitatory pools and 100 are in the inhibitory pool IH. The network also receives inputs from 800 external neurons, and these neurons increase their firing rates to apply a stimulus or distractor to one of the pools S1 or S2. Protocol S1 contains the synaptic connection matrices.

Our cortical network model features a minimal architecture to investigate stability and distractibility, and consists of two selective pools, S1 and S2 (Figure 8). We use just two selective pools to eliminate possible disturbing factors. The nonselective pool NS models the spiking of cortical neurons and serves to generate an approximately Poisson spiking dynamic in the model [21], which is what is observed in the cortex. The inhibitory pool IH contains the 100 inhibitory neurons. The connection weights between the neurons of each pool or population are called the intrapool connection strengths *w_+_*. The increased strength of the intrapool connections is counterbalanced by the other excitatory connections (*w*_−_) to keep the average input constant.

The network receives Poisson input spikes via AMPA receptors that are envisioned to originate from 800 external neurons at an average spontaneous firing rate of 3 Hz from each external neuron, consistent with the spontaneous activity observed in the cerebral cortex [41,83]. A detailed mathematical description is provided in Protocol S1.

### Analysis.

Our analysis is targeted to investigate the stability and distractibility with respect to NMDA and GABA receptor modulations. We use two different techniques: multiple trial spiking simulations and mean-field simulations. Spiking trial simulations integrate the complete neural and synaptic dynamics over time, including statistical components of the network model. Therefore, the spiking simulations are needed to assess the stability and distractibility of the dynamical system, for this depends in part on the statistical fluctuations that occur in a network of spiking neurons [85]. This is done by simulating a network configuration for several trials, each run with different random seeds, and running a statistical analysis on the data.

**Figure 9:**
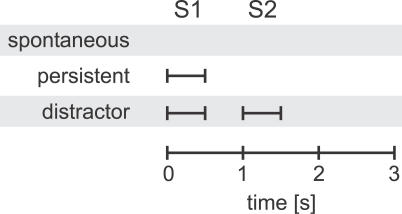
The Simulation Protocols Stimuli to either S1 or S2 are applied at different times depending on the type of simulations. The spontaneous simulations include no input. The persistent simulations assess how stably a stimulus is retained by the network. The distractor simulations add a distractor stimulus to further address the stability of the network activity.

We simulate three different conditions: the spontaneous, persistent, and distractor conditions (see Figure 9).

In spontaneous simulations, we run spiking simulations for 3 s without any extra external input. The aim of this condition is to test whether the network is stable in maintaining a low average firing rate in the absence of any inputs, or whether it falls into one of its attractor states without any external input.

In persistent simulations, an external cue of 120 Hz above the background firing rate of 2,400 Hz is applied to each neuron in pool S1 during the first 500 ms to induce a high-activity state, and then the system is run for another 2.5 s. The 2,400 Hz is distributed across the 800 synapses of each S1 neuron for the external inputs, with the spontaneous Poisson spike trains received by each synapse, thus having a mean rate of 3 Hz. The aim of this condition is to investigate whether the network, once in an attractor short-term memory state, can maintain its activity stably, or whether it falls out of its attractor, which might correspond to an inability to maintain attention.

The distractor simulations start off like the persistent simulations with a 500-ms input to pool S1 to start the S1 short-term memory attractor states, but between 1 s and 1.5 s we apply a distracting input to pool S2 with varying strengths. The aim of this condition is to measure how distractible the network is. The degree of distractibility is measured parametrically by the strength of the input to S2 required to remove the high-activity state of the S1 population. These simulation protocols serve to assess the generic properties of the dynamical attractor system rather than to model specific experimental data obtained in particular paradigms.

We used a mean-field approach (described in Protocol S1) to calculate the stationary attractor states of the network for the delay period [21]. These attractor states are independent of any simulation protocol of the spiking simulations and represent the behavior of the network by mean firing rates to which the system would converge in the absence of statistical fluctuations caused by the spiking of the neurons and by external changes. Therefore, the mean-field technique is suitable for tasks in which temporal dynamics and fluctuations are negligible. It also allows a first assessment of the attractor landscape and the depths of the basins of attraction, which then need to be investigated in detail with stochastical spiking simulations. Part of the utility of the mean-field approach is that it allows the parameter region for the synaptic strengths to be investigated to determine which synaptic strengths will on average produce stable activity in the network, for example, of persistent activity in a delay period after the removal of a stimulus. For the spontaneous state, the initial conditions for numeric simulations of the mean-field method were set to 3 Hz for all excitatory pools and 9 Hz for the inhibitory pool. These values correspond to the approximate values of the spontaneous attractors when the network is not driven by stimulus-specific inputs. For the persistent state, a selective pool was set to a higher initial value (30 Hz) to account for the excitation of these neurons during the preceding cue period.

In addition, we used the mean-field technique to assess the flow between the attractor states. The flow is the force that drives the system toward the attractor given a parameter value in phase space, i.e., the firing rates of the pools. Since we were interested in the depth of a single attractor, we used a setup with just one selective pool. This was done by fixing the value of the firing rate of the selective pool and letting the other values converge to their fixed point. Afterward, we computed the flow with this configuration [36].

## Supporting Information

Protocol S1 Neural and Synaptic Dynamics (73 KB PDF)
