# Supplementary material for: A Dynamical Systems Hypothesis of Schizophrenia
Source: PLoS Comput Biol. 2007 Nov 9;3(11):e228. doi: 10.1371/journal.pcbi.0030228 (PMC2065887; doi:10.1371/journal.pcbi.0030228)
Supplement: Supplementary file 1 [file pcbi.0030228.sd001.pdf]

Supplementary Information

Neural and synaptic dynamics

We use the mathematical formulation of the integrate-and-fire neurons and synaptic currents described in Brunel & Wang (2001). Here we provide a brief summary of this framework.

The dynamics of the sub-threshold membrane potential V of a neuron are given by the equation:

$$C_m \frac{dV(t)}{dt} = -g_m(V(t) - V_L) - I_{syn}(t), \tag{1}$$

Both excitatory and inhibitory neurons have a resting potential $V_L = -70mV$, a firing threshold $V_{thr} = -50mV$ and a reset potential $V_{reset} = -55mV$. The membrane parameters are different for both types of neurons: Excitatory (Inhibitory) neurons are modeled with a membrane capacitance $C_m = 0.5nF(0.2nF)$, a leak conductance $g_m = 25nS(20nS)$, a membrane time constant $\tau_m = 20ms(10ms)$, and a refractory period $t_{ref} = 2ms(1ms)$. Values are extracted from McCormick et al. (1985).

When the threshold membrane potential V_{thr} is reached, the neuron is set to the reset potential V_{reset} at which it is kept for a refractory period τ_{ref} and the action potential is propagated to the other neurons.

The network is fully connected with $N_E = 400$ excitatory neurons and $N_I = 100$ inhibitory neurons, which is consistent with the observed proportions of the pyramidal neurons and interneurons in the cerebral cortex (Braitenberg & Schütz 1991, Abeles 1991). The synaptic current impinging on each neuron is given by the sum of recurrent excitatory currents ($I_{AMPA,rec}$ and $I_{NMDA,rec}$), the external excitatory current($I_{AMPA,ext}$) the inhibitory current (I_{GABA}):

$$I_{syn}(t) = I_{AMPA,ext}(t) + I_{AMPA,rec}(t) + I_{NMDA,rec}(t) + I_{GABA}(t). \tag{2}$$

The recurrent excitation mediated by the AMPA and NMDA receptors, inhibition by GABA receptors. In addition, the neurons are exposed to external Poisson input spike trains mediated by AMPA receptors at a rate of 2.4kHz. These can be viewed as originating from $N_{ext} = 800$ external neurons at average rate of 3Hz per neuron, consistently with the spontaneous activity observed in the cerebral cortex (Wilson et al. 1994, Rolls & Treves 1998). The currents are defined by:

$$I_{AMPA,ext}(t) = g_{AMPA,ext}(V(t) - V_E) \sum_{j=1}^{N_{ext}} s_j^{AMPA,ext}(t) \tag{3}$$

$$I_{AMPA,rec}(t) = g_{AMPA,rec}(V(t) - V_E) \sum_{j=1}^{N_E} w_{ji}^{AMPA} s_j^{AMPA,rec}(t) \tag{4}$$

$$I_{NMDA,rec}(t) = \frac{g_{NMDA}(V(t) - V_E)}{1 + [Mg^{++}]exp(-0.062V(t))/3.57} \times \sum_{j=1}^{N_E} w_{ji}^{NMDA} s_j^{NMDA}(t) \tag{5}$$

$$I_{GABA}(t) = g_{GABA}(V(t) - V_I) \sum_{j=1}^{N_I} w_{ji}^{GABA} s_j^{GABA}(t) \tag{6}$$

where $V_E = 0$ mV, $V_I = -70$ mV, w_j are the synaptic weights, s_j's the fractions of open channels for the different receptors and g's the synaptic conductances for the different channels. The NMDA synaptic current depends on the membrane potential and the extracellular concentration of Magnesium ($[Mg^{++}] = 1$ mM, Jahr & Stevens (1990)). The values for the synaptic conductances for excitatory neurons are $g_{AMPA,ext} = 2.08$ nS, $g_{AMPA,rec} = 0.208$ nS, $g_{NMDA} = 0.654$ nS and $g_{GABA} = 2.50$ nS and for inhibitory neurons $g_{AMPA,ext} = 1.62$ nS, $g_{AMPA,rec} = 0.162$ nS, $g_{NMDA} = 0.516$ nS and $g_{GABA} = 1.946$ nS. These values are obtained from the ones used by Brunel & Wang (2001) by correcting for the different numbers of neurons. The conductances were calculated so that in an unstructured network the excitatory neurons have a spontaneous spiking rate of 3 Hz and the inhibitory neurons a spontaneous rate of 9 Hz. The fractions of open channels are described by:

$$\frac{ds_j^{AMPA,ext}(t)}{dt} = -\frac{s_j^{AMPA,ext}(t)}{\tau_{AMPA}} + \sum_k \delta(t - t_j^k) \tag{7}$$

$$\frac{ds_j^{AMPA,rec}(t)}{dt} = -\frac{s_j^{AMPA,rec}(t)}{\tau_{AMPA}} + \sum_k \delta(t - t_j^k) \tag{8}$$

$$\frac{ds_j^{NMDA}(t)}{dt} = -\frac{s_j^{NMDA}(t)}{\tau_{NMDA,decay}} + \alpha x_j(t)(1 - s_j^{NMDA}(t)) \tag{9}$$

$$\frac{dx_j(t)}{dt} = -\frac{x_j(t)}{\tau_{NMDA,rise}} + \sum_k \delta(t - t_j^k) \tag{10}$$

$$\frac{ds_j^{GABA}(t)}{dt} = -\frac{s_j^{GABA}(t)}{\tau_{GABA}} + \sum_k \delta(t - t_j^k), \tag{11}$$

where $\tau_{NMDA,decay} = 100$ ms is the decay time for NMDA synapses, $\tau_{AMPA} = 2$ ms for AMPA synapses (Hestrin et al. 1990, Spruston et al. 1995) and $\tau_{GABA} = 10$ ms for GABA synapses (Salin & Prince 1996, Xiang et al. 1998); $\tau_{NMDA,rise} = 2$ ms is the rise time for NMDA synapses (the rise times for AMPA and GABA are neglected because they are typically very short) and $\alpha = 0.5$ ms^{-1}. The sums over k represent a sum over spikes formulated as δ-Peaks $\delta(t)$ emitted by presynaptic neuron j at time t_j^k.

The equations were integrated numerically using a second order Runge-Kutta method with step size 0.02 ms. The Mersenne Twister algorithm was used as random number generator for the external Poisson spike trains and different trials for equal parameter configurations were run with different random seeds (as the only difference).

Meanfield Formulation

The meanfield approximation used in the present work was derived by Brunel & Wang (2001), assuming that the network of IF neurons is in a stationary state. In this formulation the potential of a neuron is calculated as:

$$\tau_x \frac{dV(t)}{dt} = -V(t) + \mu_x + \sigma_x \sqrt{\tau_x}\eta(t) \tag{12}$$

where $V(t)$ is the membrane potential, x labels the populations. The symbol τ_x is the effective membrane time constant, μ_x the mean value the membrane potential would have in the absence of spiking and fluctuations, σ_x measures the magnitude of the fluctuations and η

is a Gaussian process with absolute exponentially decaying correlation function with time constant τ_{AMPA}. The quantities μ_x and σ_x^2 are given by:

$$\mu_x = \frac{(T_{ext}\nu_{ext} + T_{AMPA}n_x^{AMPA} + \rho_1 n_x^{NMDA})V_E + \rho_2 n_x^{NMDA}\langle V\rangle + T_I n_x^{GABA}V_I + V_L}{S_x} \quad (13)$$

$$\sigma_x^2 = \frac{g_{AMPA,ext}^2(\langle V\rangle - V_E)^2 N_{ext}\nu_{ext}\tau_{AMPA}^2\tau_x}{g_m^2\tau_m^2}. \quad (14)$$

where $\nu_{ext} = 3$ Hz $(+\lambda_{cue})$, ν_I is the spiking rate of the inhibitory pool, $\tau_m = C_m/g_m$ with the values for the excitatory or inhibitory neurons depending of the pool considered and the other quantities are given by:

$$S_x = 1 + T_{ext}\nu_{ext} + T_{AMPA}n_x^{AMPA} + (\rho_1 + \rho_2)n_x^{NMDA} + T_I n_x^{GABA} \quad (15)$$

$$\tau_x = \frac{C_m}{g_m S_x} \quad (16)$$

$$n_x^{AMPA} = \sum_{j=1}^{p} f_j w_{jx}^{AMPA}\nu_j \quad (17)$$

$$n_x^{NMDA} = \sum_{j=1}^{p} f_j w_{jx}^{NMDA}\psi(\nu_j) \quad (18)$$

$$n_x^{GABA} = \sum_{j=1}^{p} f_j w_{jx}^{GABA}\nu_j \quad (19)$$

$$\psi(\nu) = \frac{\nu\tau_{NMDA}}{1+\nu\tau_{NMDA}}\left(1 + \frac{1}{1+\nu\tau_{NMDA}}\sum_{n=1}^{\infty}\frac{(-\alpha\tau_{NMDA,rise})^n T_n(\nu)}{(n+1)!}\right) \quad (20)$$

$$T_n(\nu) = \sum_{k=0}^{n}(-1)^k\binom{n}{k}\frac{\tau_{NMDA,rise}(1+\nu\tau_{NMDA})}{\tau_{NMDA,rise}(1+\nu\tau_{NMDA}) + k\tau_{NMDA,decay}} \quad (21)$$

$$\tau_{NMDA} = \alpha\tau_{NMDA,rise}\tau_{NMDA,decay} \quad (22)$$

$$T_{ext} = \frac{g_{AMPA,ext}\tau_{AMPA}}{g_m} \quad (23)$$

$$T_{AMPA} = \frac{g_{AMPA,rec}N_E\tau_{AMPA}}{g_m} \quad (24)$$

$$\rho_1 = \frac{g_{NMDA}N_E}{g_m J} \quad (25)$$

$$\rho_2 = \beta\frac{g_{NMDA}N_E(\langle V_x\rangle - V_E)(J-1)}{g_m J^2} \quad (26)$$

$$J = 1 + \gamma\exp(-\beta\langle V_x\rangle) \quad (27)$$

$$T_I = \frac{g_{GABA}N_I\tau_{GABA}}{g_m} \quad (28)$$

$$\langle V_x\rangle = \mu_x - (V_{thr} - V_{reset})\nu_x\tau_x, \quad (29)$$

where p is the number of excitatory pools, f_x the fraction of neurons in the excitatory x pool, $w_{j,x}$ the weight of the connections from pool x to pool j, ν_x the spiking rate of the x excitatory pool, $\gamma = [Mg^{++}]/3.57$ and $\beta = 0.062$.

The spiking rate of a pool as a function of the defined quantities is then given by:

$$\nu_x = \phi(\mu_x, \sigma_x), \tag{30}$$

where

$$\phi(\mu_x, \sigma_x) = \left(\tau_{rp} + \tau_x \int_{\beta(\mu_x,\sigma_x)}^{\alpha(\mu_x,\sigma_x)} du\sqrt{\pi}\exp(u^2)[1 + \text{erf}(u)]\right)^{-1} \tag{31}$$

$$\alpha(\mu_x, \sigma_x) = \frac{(V_{thr} - \mu_x)}{\sigma_x}\left(1 + 0.5\frac{\tau_{AMPA}}{\tau_x}\right) + 1.03\sqrt{\frac{\tau_{AMPA}}{\tau_x}} - 0.5\frac{\tau_{AMPA}}{\tau_x} \tag{32}$$

$$\beta(\mu_x, \sigma_x) = \frac{(V_{reset} - \mu_x)}{\sigma_x} \tag{33}$$

where erf(u) the error function and τ_{rp} the refractory period which is considered to be 2 ms for excitatory neurons and 1 ms for inhibitory neurons. To solve the equations defined by (30) for all x we integrate numerically (29) and the differential equation below, which has fixed point solutions corresponding to equations 30:

$$\tau_x \frac{d\nu_x}{dt} = -\nu_x + \phi(\mu_x, \sigma_x). \tag{34}$$

The equations were integrated using the Euler method with step size 0.2 and 8000 iterations, which allowed for convergence.

Connection Matrices

Fraction of pool sizes f_i

Values are relative to all neurons, not only the excitatory portion.

S1	S2	NS	IH
0.08	0.08	0.64	0.2

Connection matrix for AMPA and NMDA – [from, to]

	S1	S2	NS	IH
S1	w_+	w_-	1	1
S2	w_-	w_+	1	1
NS	w_-	w_-	1	1
IH	0	0	0	0

where $w_- = \frac{0.8 - f_{S1}w_+}{0.8 - f_{S1}}$.

Connection matrix for GABA – [from, to]

	S1	S2	NS	IH
S1	0	0	0	0
S2	0	0	0	0
NS	0	0	0	0
IH	1	1	1	1

References

Abeles, M. (1991), *Corticonics*, Cambridge University Press, New York.

Braitenberg, V. & Schütz, A. (1991), *Anatomy of the Cortex*, Springer Verlag, Berlin.

Brunel, N. & Wang, X. (2001), 'Effects of neuromodulation in a cortical network model of object working memory dominated by recurrent inhibition', *Journal of Computational Neuroscience* **11**, 63–85.

Hestrin, S., Sah, P. & Nicoll, R. (1990), 'Mechanisms generating the time course of dual component excitatory synaptic currents recorded in hippocampal slices', *Neuron* **5**, 247–253.

Jahr, C. & Stevens, C. (1990), 'Voltage dependence of NMDA-activated macroscopic conductances predicted by single-channel kinetics', *Journal of Neuroscience* **10**, 3178–3182.

McCormick, D., Connors, B., Lighthall, J. & Prince, D. (1985), 'Comparative electrophysiology of pyramidal and sparsely spiny stellate neurons in the neocortex', *Journal of Neurophysiology* **54**, 782–806.

Rolls, E. T. & Treves, A. (1998), *Neural Networks and Brain Function*, Oxford University Press, Oxford.

Salin, P. & Prince, D. (1996), 'Spontaneous GABA-A receptor mediated inhibitory currents in adult rat somatosensory cortex', *Journal of Neurophysiology* **75**, 1573–1588.

Spruston, N., Jonas, P. & Sakmann, B. (1995), 'Dendritic glutamate receptor channel in rat hippocampal CA3 and CA1 pyramidal neurons', *Journal of Physiology* **482**, 325–352.

Wilson, F., Scalaidhe, S. & Goldman-Rakic, P. (1994), 'Functional synergism between putative gamma-aminobutyrate-containing neurons and pyramidal neurons in prefrontal cortex', *Proceedings of the National Academy of Science* **91**, 4009–4013.

Xiang, Z., Huguenard, J. & Prince, D. (1998), 'GABA-A receptor mediated currents in interneurons and pyramidal cells of rat visual cortex', *Journal of Physiology* **506**, 715–730.
